# Ratiometric optical dual gas sensor for simultaneous detection of nitric oxide and oxygen intended for healthcare, biological, and biomedical applications

**DOI:** 10.1039/d4ra07017h

**Published:** 2024-12-02

**Authors:** Rispandi, Cheng-Shane Chu, Sri Nugroho, Muhammad Imam Ammarullah

**Affiliations:** a Department of Mechanical Engineering, Faculty of Engineering, Universitas Andalas Padang 25163 West Sumatra Indonesia; b Department of Mechanical Engineering, Ming Chi University of Technology New Taipei City 24301 Taiwan; c Research Center for Intelligent Medical Device, Ming Chi University of Technology New Taipei City 24301 Taiwan; d International PhD Program in Innovative Technology of Biomedical Engineering and Medical Devices, Ming Chi University of Technology New Taipei City 24301 Taiwan; e Department of Mechanical Engineering, Faculty of Engineering, Universitas Diponegoro Semarang 50275 Central Java Indonesia imamammarullah@gmail.com; f Undip Biomechanics Engineering & Research Centre (UBM-ERC), Universitas Diponegoro Semarang 50275 Central Java Indonesia

## Abstract

Developing efficient and reliable gas sensors for the simultaneous detection of multiple gases is paramount in various fields, specifically healthcare, biological, and biomedical applications. In this study, we present a novel ratiometric optical dual gas sensor based on perovskite quantum dots (PQDs) to detect nitric oxide and oxygen simultaneously. All dye molecules were excited using a 405 nm LED in the experimental setup. The results obtained from the experiments reveal that both the optical NO and O_2_ sensors exhibit linear Stern–Volmer plots, and the sensitivities observed for NO and O_2_ sensors were approximately 2.4 and 2.8, respectively. The response and recovery times of the optical NO sensor are 67 s and 69 s, respectively. O_2_ sensor response and recovery times are 66 s and 68 s, respectively. In this work, the ratiometric optical dual gas sensing approach suppressed the effects of spurious fluctuations in the intensity of external and excitation sources.

## Abstract 2

Developing efficient and reliable gas sensors for the simultaneous detection of multiple gases is paramount in various fields, specifically healthcare, biological, and biomedical applications.

## Graphical abstract

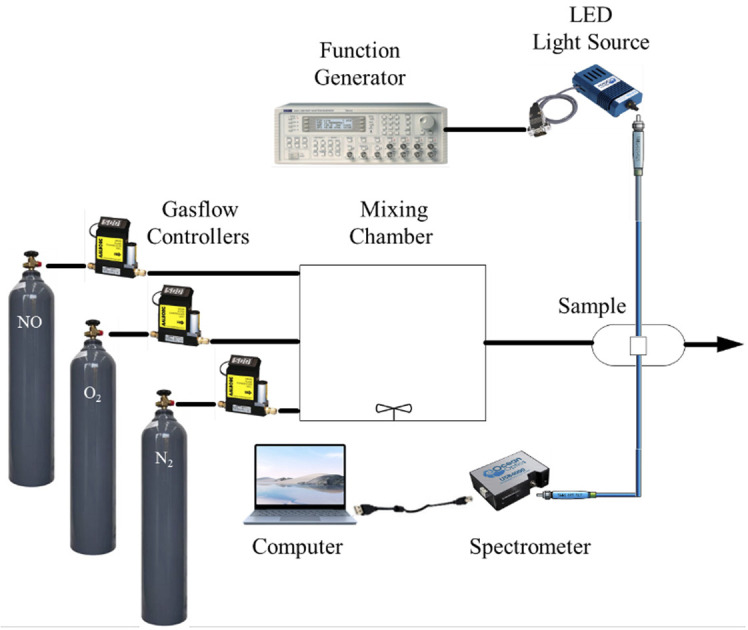

## 1 Introduction

In recent years, there has been a growing interest in developing optical dual gas sensors due to their significant potential in various applications, particularly in healthcare, biological, and biomedical fields. These sensors can simultaneously detect two gases, which is crucial for monitoring complex biological processes^1^ and environmental conditions.^2^ Among the various gases of interest, nitric oxide (NO)^3^ and oxygen (O_2_)^4^ are particularly important due to their critical roles in physiological and pathological processes. Optical dual gas sensors leverage the unique optical properties of specific materials that change in response to the presence of target gases. These sensors typically employ luminescent or colorimetric indicators that provide a measurable optical signal when exposed to the gases of interest.^5–9^ The dual gas sensing capability is achieved using two distinct indicators, each selective to one of the target gases. One of the primary advantages of optical sensors is their ability to provide real-time, non-invasive, and susceptible measurements. This makes them particularly suitable for applications where rapid and accurate detection is essential. Furthermore, optical sensors can be miniaturized and integrated into portable devices, enhancing their applicability in point-of-care diagnostics and wearable health monitoring systems. A ratiometric optical dual gas sensor is a device that can detect two gases simultaneously by measuring the ratio of two optical signals. In this case, the sensor is designed to detect nitric oxide and oxygen. The sensor works by using two different indicators that respond to the two gases, and the ratio of the signals from the two indicators is used to determine the concentrations of the gases. The fusion of optical sensing principles with the unique material properties of optical dual gas sensors opens up new avenues for real-time monitoring and analysis, addressing critical challenges in healthcare^10–12^ and biomedical applications.^13–17^

Nitric oxide (NO) is a signaling molecule involved in numerous physiological processes, including vasodilation, neurotransmission, and immune response. Abnormal NO levels are associated with various health conditions, such as cardiovascular diseases, neurodegenerative disorders, and inflammation. Therefore, accurate monitoring of NO levels is essential for early diagnosis and management of these conditions. Nitric oxide is a colorless, harmful, and toxic gas. However, it is vital for regulating circulation in the human body. Sensing nitric oxide molecules is fundamentally essential in elucidating human cell functionality, pathology, and toxicity, and it is also practical in developing potential platforms for medical and environmental applications.^18–23^ Nitric oxide is a molecule that has recently attracted heightened interest due to its vast role in human health and biology. In particular, NO is released by various cells in mammalian systems and plays a vital role in many biological processes, including regulating cell function in the nervous, vascular, and immune systems, neurotransmission, vasodilation, blood pressure, *etc.*^24–31^

Oxygen (O_2_), on the other hand, is vital for cellular respiration and energy production in living organisms. Monitoring O_2_ levels is crucial in medical settings, especially critical care, anesthesia, and respiratory therapy. Hypoxia (low oxygen levels) and hyperoxia (high oxygen levels) can lead to severe complications and must be carefully managed. Oxygen is a colorless and odorless gas essential to human life for respiration, the environment, the oceans, agriculture, industry, and health. An oxygen concentration range of 19.5–23.5% in the environment is vital for living life.^32^ In addition, monitoring oxygen levels is paramount across various domains, including food packaging, chemical, environmental, and biomedical technology. An optical gas sensor can gauge oxygen concentration by observing the reduction in the fluorescence intensity of a dye molecule through its interaction with oxygen, a phenomenon known as quenching. The reported optical sensors employ a fluorescence indicator embedded in solid matrices, such as a polymer,^33–36^ sol–gel matrix,^37–39^ or electrospun fiber,^40^ for hosting oxygen-sensitive fluorophores and assisting oxygen in penetrating supporting matrices. Therefore, oxygen-sensitive fluorescent dyes' properties and supporting matrices are essential for fluorescence-based optical sensors. Many studies have suggested ethyl cellulose (EC) as a matrix for detecting the O_2_ gas.^41^ On the other hand, 3D printing has recently become an innovative, versatile, and cost-effective technique.^42–44^ It allows for the simultaneous integration of multiple fluorescent dyes into a silicone matrix. Significant efforts have been made to develop reliable methods for evaluating ratiometric optical dual gas sensors for nitric oxide and oxygen.

This work presents a ratiometric optical dual gas sensor for simultaneous detection of NO and O_2_ gas sensors. The supporting matrixes significantly influence the performance of fluorescence-based optical sensors. Numerous studies have recommended using the EC and silicone matrix due to its notable multifunction gas permeability, robust mechanical and chemical stability, and excellent optical transparency in UV LED. In this case, the optical dual gas sensor used a new material dye of perovskite QDs (PQDs). CsPbBr_3_ perovskite QDs embedded in the EC matrix emerge as a promising material for a novel optical nitric oxide sensor.

Meanwhile, FAPbI_3_ perovskite QDs use the silicone matrix for the oxygen sensor. All sensitivity dyes are coated on filter paper to detect the gas NO–O_2_ sensors on the upper and bottom surfaces of the device. The wavelength spectrum emission of an optical dual sensor sensitive to the selective gases was detected simultaneously. The cost and durability of the materials used for sensing with perovskite quantum dots (PQDs) are much cheaper based on Perovskite QDs materials. Because it is more accessible to manufacture with fewer fabrication steps, affordable for low-to medium-volume applications, and the sensor can be used more than once. Perovskite QDs like CsPbBr_3_ and FAPbI_3_ are relatively cost-effective to synthesize compared to traditional gas sensing materials, making them a promising choice for cost-sensitive applications. However, perovskite materials are sensitive to moisture and light exposure, impacting their durability. Encapsulation in protective matrices such as ethyl cellulose (EC) and silicone enhances stability, improving durability significantly and potentially allowing the sensor to function reliably over several months in controlled environments. The newly developed perovskite quantum dots (PQDs) sensing material is intended for healthcare, biological, and biomedical applications. Table 1 compares materials used for optical nitric oxide and oxygen sensors, highlighting the differences in characteristics between standard optical sensors and various sensor types made from different nitric oxide and oxygen-sensitive materials.

**Table 1:** Comparisons properties of the optical dual gas sensors for simultaneous detection of NO–O_2_ gas sensors

NO probe	O_2_ probe	Support matrix	Range	Response time	Sensitivity	Method/type	Ref.
CsPbBr_3_ perovskite QDs	PtTFPP	Cellulose acetate	NO: 0–1000 ppm	NO: 71 s/109 s	NO: 2.7	Single electrospun fibers/intensity	41
			O_2_: 0–100%	O_2_: 60 s/65 s	O_2_: 10.7		
Ag/AgCl	Pt	Phosphate-buffered saline	NO: <5 nM	NO: −0.4 V	NO: 51.0 ± 14.2 pA μm^−1^	Dual electrochemical/current	45
			O_2_: <500 nM	O_2_: +0.75 V	O_2_: 178.1 ± 82.1 pA μm^−1^		
Ag/AgCl	Pt	Phosphate-buffered saline	NO: 3.6 (±0.9)	NO: 50 ± 10 s	NO: 45.2 pA μm^−1^	Dual electrochemical/current	46
			O_2_: 0.41 (±0.04)	O_2_: 30 ± 10 s	O_2_: 172.3 pA μm^−1^		
CsPbBr_3_ QDs	FAPbI_3_ QDs	Ethyl cellulose silicone	NO: 0–1000 ppm	NO: 67 s/69 s O_2_: 66 s/68 s	NO: 2.4	Dual sensor/ratiometric intensity	This work
			O_2_: 0–100%		O_2_: 2.8		

## 2 Materials and methods

### 2.1 Materials

CsPbBr_3_ and FAPbI_3_ perovskite quantum dots (PQDs) were made using a reference-based synthetic material used as a sensitive nitric oxide and oxygen dye. Oxazine 170 perchlorate (95%) was purchased from Aldrich and used as the reference signal. Ethyl cellulose (EC) was purchased from Tokyo Chemical Industry Co., Ltd (TCI) (Chuo, Japan), and silicone was purchased from San Draw Inc. (Taichung City, Taiwan) and used as the matrix. Other reagents, such as tetrahydrofuran (THF, 99.9%) and toluene (99.8%), were purchased from TEDIA (Fairfield, CT, USA). EtOH (99.5%) was purchased from ECHO Chemical Co. Ltd (Miaoli, Taiwan). All the chemicals were used readily in the experiment without purification.

**Fig. 1:**
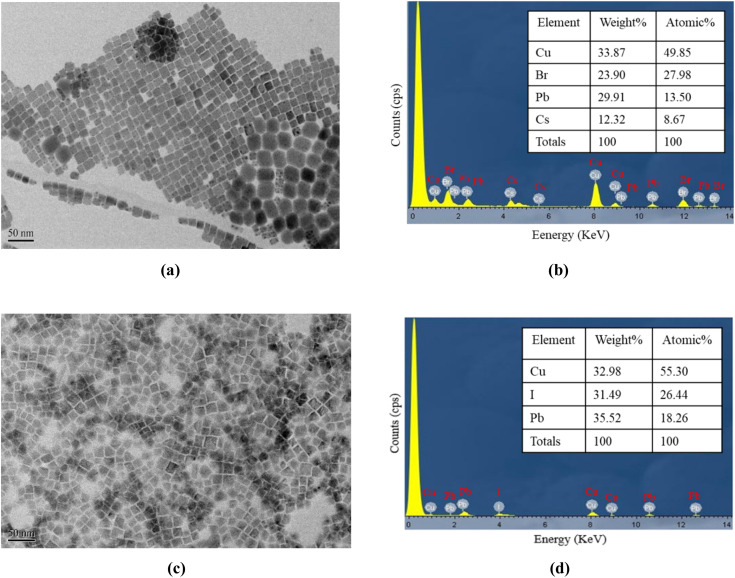
(a) TEM images and (b) EDS spectra of CsPbBr_3_ perovskite QDs, (c) TEM images and (d) EDS analysis result for FAPbI_3_ perovskite QDs.

Fig. 1a shows a transmission electron microscopy (TEM) image of the CsPbBr_3_ perovskite QDs at resolutions of 50 nm. Fig. 1b presents the energy-dispersive X-ray spectroscopy (EDS) outcome for the CsPbBr_3_ perovskite QDs, revealing the presence of atomic Cs, Pb, Br, and Cu. On the other hand, Fig. 1c illustrates a planar SEM image of the FAPbI_3_ perovskite QDs at a resolution of 50 nm, which shows highly dense and smooth morphology without aggregates. The energy dispersive spectroscopy (EDS) technique was used to qualitatively analyze FAPbI_3_ perovskite QDs films and detect the presence of atomic I, Pb, and Cu, as shown in Fig. 1d. The *y*-axis corresponds to the count per second per electron, representing X-ray intensity, while the *x*-axis represents kilo-electron volts (keV) energy. The copper content is attributed to the presence of the copper grid.

### 2.2 Methods

#### 2.2.1 Synthesis of CsPbBr_3_ perovskite quantum dots

The method outlined in ref. 47 synthesized CsPbBr_3_ quantum dots (QDs). In summary, 200 mg of cesium carbonate (99.99%) was placed in a 25 mL three-neck flask along with 9 mL of octadecene (ODE) (90%) and 0.75 mL of oleic acid (OA) (90%). The mixture was then stirred on a hot plate at 150 °C for 30 minutes until a clear solution formed. Separately, a reaction solution was prepared by dissolving 0.09 M of PbBr_2_ in 30 mL of ODE, 3 mL of OA, and 3 mL of octylamine, which was also stirred on a hot plate at 120 °C for 30 minutes until transparent. Following this, a 0.8 mL precursor solution was swiftly added to the reaction solution using a hot-injection method, and the resultant mixture was transferred to a glass vial submerged in an ice-water bath to yield unpurified CsPbBr_3_ QDs solutions.

#### 2.2.2 Synthesis of FAPbI_3_ perovskite quantum dots

The procedure described in ref. 48 was employed to synthesize FAPbI_3_ QDs and post-treatment with PEAI. Formamidine acetate (0.521 g; FA-acetate 99%, Aldrich) and OA (10 mL; 90%; Sigma-Aldrich) were placed in a flask and degassed by heating at 120 °C for one hour and then stirred at 80 °C for one hour under N_2_ to prepare the FA-oleate precursor. PbI_2_ (175 mg; 99.999%; Sigma-Aldrich) and octadecene (10 mL; ODE; 90%; Sigma-Aldrich) were placed in a three-necked flask and degassed by heating at 100 °C for one hour, and after that, the temperature was increased to 120 °C under N_2_. OAm (1 mL; 90%; Sigma-Aldrich) and OA (1 mL) were injected into the flask at 120 °C. After the PbI_2_ salt was completely dissolved, the solution was cooled to 80 °C. Subsequently, 1 mL of FA-acetate precursor was injected. After 5 s, the reaction flask was cooled in an ice bath to obtain the FAPbI_3_ crude solution. For pristine FAPbI_3_ QDs (PEAI0), the as-prepared natural solution was subjected to a purification process. The solution was centrifuged at 12 000 rpm for 10 minutes in the first step. The obtained residue was then added to 7 mL hexane (95% Sigma-Aldrich) and 7 mL ethyl acetate (99% Alfa-Aesar) (1 : 1 v/v ratio) and centrifuged at 12 000 rpm for 15 min. The precipitate was collected, dispersed in 2 mL octane (98+%; Alfa Aesar), and centrifuged at 12 000 rpm for 15 min. Finally, the supernatant was stored at 4 °C until further use.

#### 2.2.3 Preparation of optical NO and O_2_ sensing materials

Nitric oxide material sensing using a supporting matrix was prepared by dissolving 1.25 g of EC in 10 mL of toluene and 2.25 mL of EtOH (99.5%) as described elsewhere in ref. 49. The solution was capped and stirred magnetically until it was turned into a transparent glue-like state. Furthermore, 2 mg of oxide 170 (O170) dye was dissolved in 10 mL of tetrahydrofuran (THF 99.9%) to produce a reference signal, and then 20 μL of the synthesized CsPbBr_3_ perovskite QDs solution. The last, nitric oxide-sensitive material was prepared by mixing the 10 μL EC matrix, 5 μL O170/THF solutions, and 20 μL of the synthesized CsPbBr_3_ perovskite QDs solution, dried for 10 min at room temperature.

**Fig. 2:**
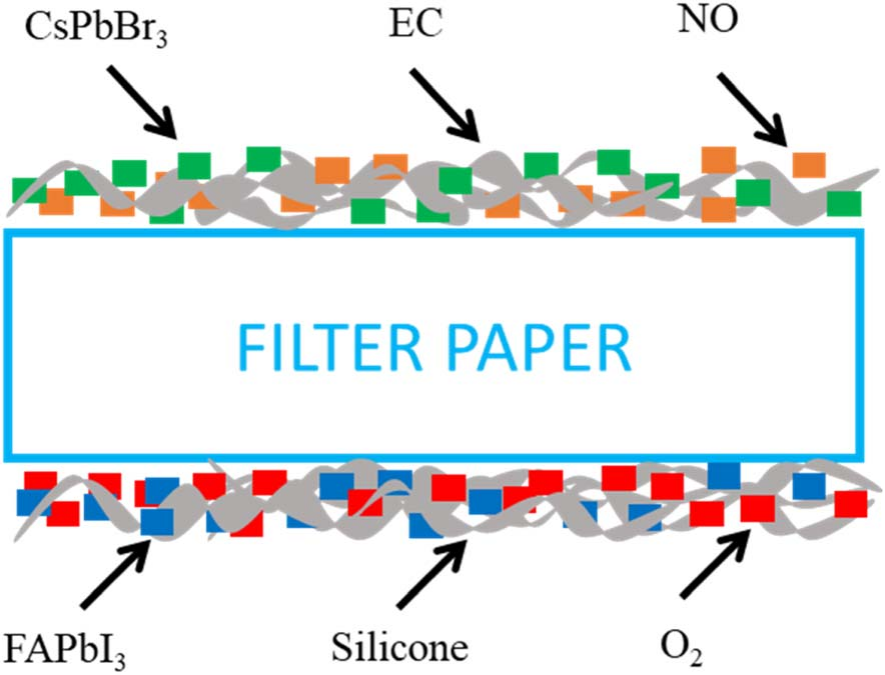
Schematic structure device sensing of the NO–O_2_ dual gas sensor.

On the other hand, in the next steps of the O_2_ sensing material, the supporting matrix of the oxygen sensor was formulated using 150 mg of silicone. The O_2_ sensitivity indicator solution was prepared with 0.5 μL of the synthesized FAPbI_3_ perovskite QDs solution using the same reference signal solution of 10 μL of oxide 170 (O170). The last oxygen-sensitive material was prepared by mixing 0.5 μL of FAPbI_3_ perovskite QDs, 10 μL of oxide 170 (O170)/THF, and 150 mg of silicone as a material sensing to detect oxygen at room temperature. Both sensing materials' nitric oxide and oxygen were coated on the top and bottom of filter paper, as shown schematically in Fig. 2.

### 2.3 Instruments

**Fig. 3:**
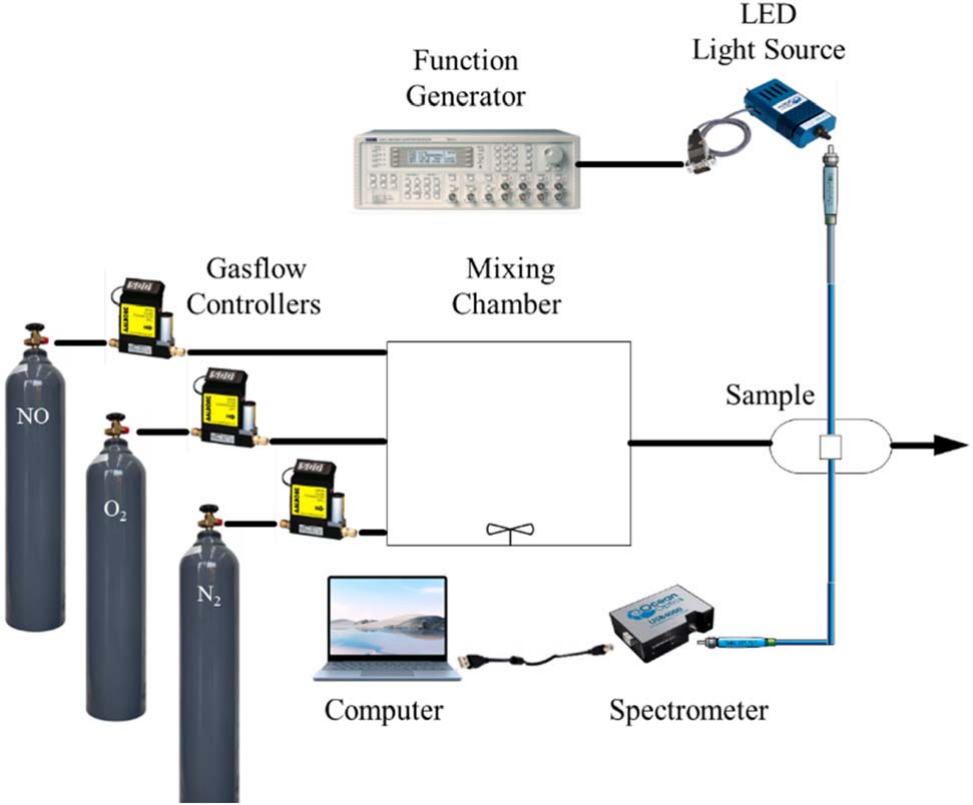
Schematic diagram gas sensing used for characterization.

NO, O_2_, and N_2_ gas concentrations in the gas flow are controlled using mass flow controls (Aalborg Instruments and Controls Inc., New York, NY, USA, Model GFC 17) and used to control the flow rate of the gas entering the system, ensuring a stable and repeatable gas concentration. It contains the target gas for testing (NO, O2, and N2). The gases are in a mixing chamber and blended before being sent to the sample holder. Ensures a homogenous mixture of the target and carrier gases. To prevent interference from outside light, the sample holder is appropriately insulated. It is often sealed and can be vacuum-sealed, controlling humidity and temperature for accuracy. Next, the sensor is connected to data acquisition systems, measuring the change in spectrum signal. The spectrum signal of the optical dual gas sensor was excited by a 405 nm UV LED light source (Ocean Optics, Series LS-450), driven by a wave function generator with a pulse signal at a frequency of 10 kHz function generator waveform (Version TGA1240). Records sensor output in real-time, often connected to software for data analysis. The fluorescence spectra of the optical dual sensor were recorded using a USB 4000 spectrofluorometer. A USB4000 fiber optic spectrometer has been attached *via* the holder to one side of the sample, which measures the fluorescence intensities given off by various fluorophores. The SpectraSuite software, which has a 10 ms integration time, is used to show the detected intensity data on a computer. Allows control, real-time monitoring, and data analysis with a graphical representation of the gas response curves. The intensity data is copied into the MS Excel program and then moved to the original program for graphing. Ensures that gases are safely released or neutralized after the test, following safety and environmental standards. The schematic diagram of gas sensing used for the optical dual sensor is shown in Fig. 3. This schematic diagram provides a step-by-step approach to gas sensing characterization, ensuring precise, controlled measurements and safe handling of gases.

## 3 Results and discussions

### 3.1 Optical properties of optical dual sensor

**Fig. 4:**
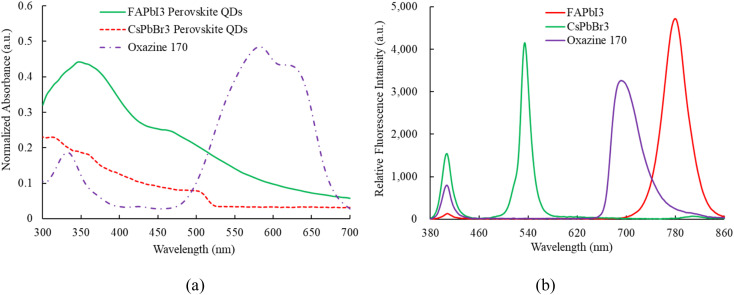
(a) Absorption and (b) emission spectrum of FAPbI_3_, CsPbBr_3_ QDs, and oxazine 170.

Fig. 4a illustrates the absorption results, where sensing materials incorporating FAPbI_3_, CsPbBr_3_, and oxazine 170 (O170) as reference fluorescent dyes are employed to detect NO and O_2_ simultaneously. All sensing materials are illuminated using a 405 nm UV LED. The absorption spectra of each sensing material are individually recorded by the optical dual sensor, which combines the emission spectra of perovskite CsPbBr_3_ QDs and FAPbI_3_ QDs. Specifically, the absorption peaks for perovskite FAPbI_3_, CsPbBr_3_, and oxazine 170 are observed at approximately 450, 480, and (500–650) nm, respectively. Calculating the band gap from the absorption spectra is crucial for understanding the sensing mechanisms of materials like CsPbBr_3_ and FAPbI_3_. The band gap determines the energy levels at which electrons can be excited and relaxed, directly influencing the optical and electronic properties essential for sensing applications. The device sensor of the CsPbBr_3_ and FAPbI_3_ by extrapolating the linear part of the (*αhν*)^2^*versus* the photon–energy plot optical bandgaps is estimated to be 2.3 eV and 1.50 eV, respectively. Fig. 4b shows the room-temperature emission spectrum of the ratiometric optical dual sensor using perovskite FAPbI_3_, CsPbBr_3_, and O170 as reference signals. It can be seen that they exhibit an intense emission spectrum at 770 nm, 520 nm, and 650 nm, respectively. Notably, the emission signals from perovskite FAPbI_3_, CsPbBr_3_ QDs, and O170 do not overlap. Finally, the emission spectrum of the sensing material under investigation will undergo further scrutiny in the presence of optical NO and O_2_ dual sensors.

### 3.2 Nitric oxide sensing properties of optical dual sensor

**Fig. 5:**
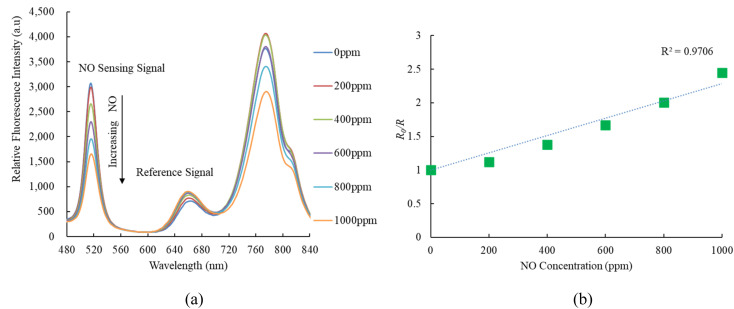
(a) Response of optical dual sensor at different NO concentrations and (b) Stern–Volmer plot.

Fig. 5a illustrates the emission spectrum of the optical dual sensor concerning various concentrations of nitric oxide. The dual sensor was excited by a 405 nm LED, and the emission spectrum of CsPbBr_3_ perovskite QDs with a peak wavelength of 520 nm is highly sensitive to nitric oxide concentration. In contrast, oxygen gas impacts the fluorescence signals of CsPbBr_3_ perovskite QDs. However, this effect does not extend to the nitric oxide sensor, indicating optical dual-sensor selectivity for nitric oxide sensing. The findings validate that introducing nitric oxide molecules induces a quenching effect on the fluorescence emitted by CsPbBr_3_ perovskite QDs. This intentional quenching process leads to a noteworthy reduction in the emission spectrum of CsPbBr_3_ perovskite QDs, with the reduction becoming more pronounced as nitric oxide concentrations increase from 0 to 1000 ppm. Notably, the intensities of the other fluorescence signals are unaffected or exhibit a negligible effect in the presence of nitric oxide, facilitating the detection of oxygen without any interference. *R*_0_ and *R* can evaluate the response of the ratiometric optical sensor in the Stern–Volmer equation:^50^

1 *I*/*I*_0_ = [*f*/1 + *K*_SV_[*Q*] + (1 − *f*)]^−1^

*I*_0_ and *I* represent the steady-state fluorescence intensities in the absence and presence of the quencher molecules, respectively; *K*_sv_ is the Stern–Volmer quenching constant; and [*Q*] is the concentration of the quencher molecules; and *f* is the fraction of the fluorescence caused by the sensitive molecules in a quencher-free environment.

In the ratiometric optical dual sensor developed in this study, the NO–O_2_ concentration is derived from the ratio of the maximum fluorescence intensity of CsPbBr_3_ and FAPbI_3_ perovskite QDs to that of oxazine 170 reference fluorescent dye, *i.e.*

2 *R* = *I*_CsPbBr_3__ (520 nm)*I*_FAPbI_3__ (770 nm)/*I*_O170_ (650 nm)

where, *I*_CsPbBr_3__ (520 nm), *I*_FAPbI_3__ (770 nm), and *I*_O170_ (650 nm) represent the steady-state fluorescence intensities of the CsPbBr_3_, FAPbI_3_ perovskite QDs, and O170 respectively. The response of the ratiometic optical dual gas sensor can be calculated by replacing *I*_0_ and *I* in the Stern–Volmer equation by *R*_0_ and *R*, respectively, *i.e.*

3 *R*_0_/*R* = [*f*/1 + *K*_SV_[*Q*] + (1 − *f*)]^−1^

*R*_0_ and *R* represent the ratio of fluorescence intensity sensing signal in the absence and presence of the quencher molecules, respectively. The sensitivity of the optical dual gas sensor is defined as the ratio *R*_0_/*R*, where *R*_0_ and *R* represent the ratio of fluorescence intensities in pure nitrogen, nitric oxide and oxygen environments, respectively.

On the other hand, Fig. 5b presents the Stern–Volmer plot for the optical nitric oxide sensor employing CsPbBr_3_ perovskite QDs. The Stern–Volmer plot illustrates the relationship between nitric oxide and the ratio of relative fluorescence intensity to sensitivity (*R*_0_/*R*) concerning nitric oxide concentrations in the optical dual sensor. The optical dual gas sensor achieves maximum sensitivity to nitric oxide gas molecules, reaching 2.4 in a 1000 ppm NO, and the Stern–Volmer plot result is linear. Sensor sensitivity is the ratio *R*_0ppmNO_/*R*_1000ppmNO_, where *R*_0ppmNO_ and *R*_1000ppmNO_ represent measured fluorescence intensities in 100% N_2_ and 1000 ppm NO gases, respectively.

### 3.3 Oxygen sensing properties of optical dual sensor

**Fig. 6:**
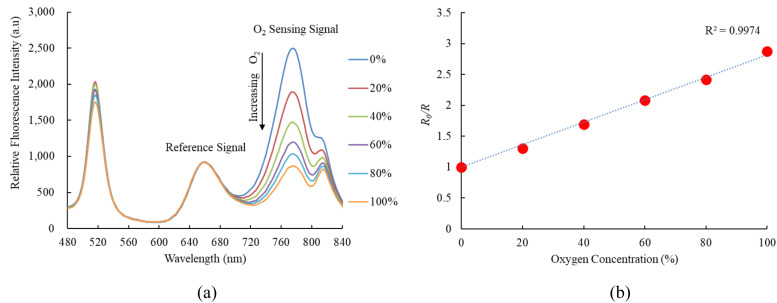
(a) Response of optical dual sensor at different O_2_ concentrations and (b) Stern–Volmer plot.

Fig. 6a indicates the response of FAPbI_3_ perovskite QDs as an oxygen sensor across varying oxygen concentrations from 0% to 100%, revealing strong fluorescence spectra at 770 nm. The relative fluorescence intensity of the FAPbI_3_ perovskite QDs oxygen sensor decreases as the oxygen concentration increases from 0% to 100%. Results further demonstrate that the sensitivity of the optical oxygen sensor is 2.8, with a linear representation in the Stern–Volmer plot shown in Fig. 6b. The sensitivity of the optical oxygen sensor is defined as the ratio *R*_0%O_2__/*R*_100%O_2__, where *R*_0%O_2__ and *R*_100%O_2__ represent the measured fluorescence intensities in 100% N_2_ and 100% O_2_ gases, respectively. The relationship between *R*_0_ and *R* under different oxygen concentration mirrors observed in the optical nitric oxide sensor can be elucidated using eqn (1). Additionally, we conducted a sensitivity check of FAPbI_3_ perovskite QDs for oxygen sensing, comparing it with the optical dual sensor. The results were comparable to this study based on the optical dual gas sensor for oxygen sensing, revealing no significant differences.

### 3.4 Response time of the optical dual sensor

**Fig. 7:**
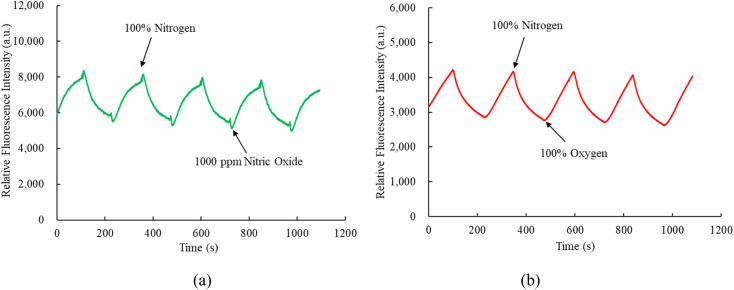
Response and recovery time of the optical dual sensor for (a) NO and (b) O_2_ sensor.

Fig. 7a and b depict the nitric oxide and oxygen response times of optical dual sensors utilizing sensitive dyes CsPbBr_3_ and FAPbI_3_ perovskite QDs. In Fig. 7a, the response and recovery times for the optical nitric oxide sensor are 67 s and 69 s, respectively, observed during the transition from 100% N_2_ to 1000 ppm NO and the subsequent recovery from 1000 ppm NO to 100% N_2_. Conversely, Fig. 7b demonstrates a 66 s response time for the optical oxygen sensor when transitioning from 100% N_2_ to 100% O_2_, with a recovery time of 68 s from 100% O_2_ to 100% N_2_. This response and recovery cycle was repeated 5 cycles, with nitric oxide and oxygen gases opened for 2 minutes until the fluorescence intensity reached a saturation value, indicating a stable and reproducible environmental signal provided by CsPbBr_3_ and FAPbI_3_ perovskite QDs. The proposed optical dual sensor method exhibits swift responsiveness and is suitable for real-world applications.

### 3.5 Dynamic response of the optical dual sensor

**Fig. 8:**
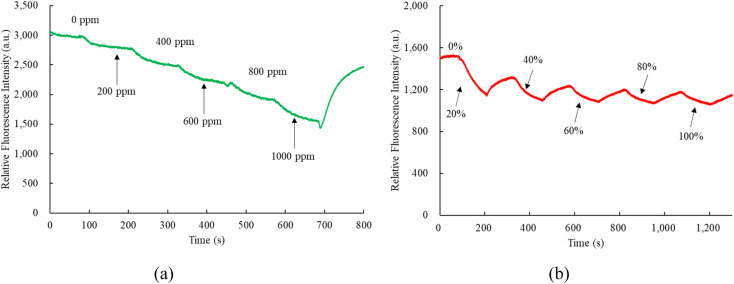
Dynamic response of optical dual sensor for (a) NO and (b) O_2_ sensor.

Observing the dynamic response times of perovskite CsPbBr_3_ and FAPbI_3_ incorporated into matrix EC and silicone revealed a comparable pattern to the response time obtained from the optical dual sensor, as depicted in Fig. 8. Fig. 8a presents the transition of the optical dual sensor for nitric oxide detection, shifting from 100% N_2_ to 1000 ppm NO, showcasing a typical dynamic response. The concentration of nitric oxide gas increased gradually at 125 s intervals during the recovery of fluorescence intensity under a 100% N_2_ atmosphere. Similarly, Fig. 8b illustrates a dynamic response when altering oxygen concentrations from 100% N_2_ to 100% O_2_. The oxygen gas concentration increased incrementally at 120 s intervals during the recovery of fluorescence intensity under a 100% N_2_ atmosphere.

### 3.6 Photostability of the optical dual sensor

**Fig. 9:**
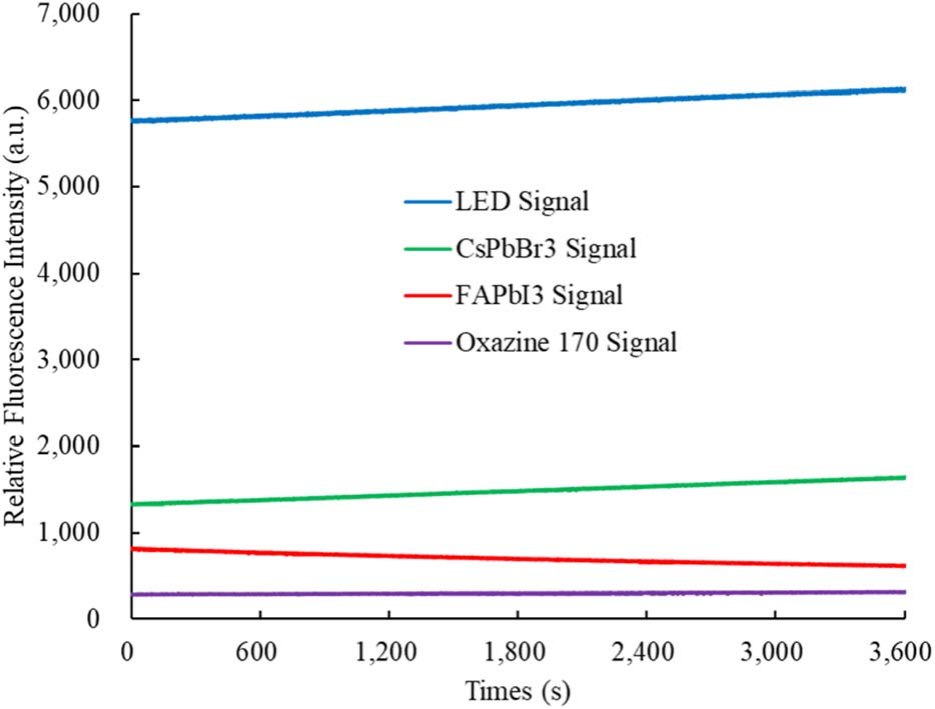
Photostability of the optical dual sensor.

Photostability was evaluated by stimulating the perovskite CsPbBr_3_, FAPbI_3_, and oxazine 170-sensitive dyes using a 405 nm UV LED light source for over 1 hour at room temperature in the ambient environment. The change in fluorescence spectrum intensity was monitored using a spectrometer instrument. Fig. 9 shows the photostability of the fluorescence intensity of the perovskite CsPbBr_3_, FAPbI_3_, and oxazine 170, which are around 1325 ± 82, 815 ± 43, and 284 ± 04, respectively. In this experiment, we have investigated that the photostability of the optical dual sensor does not change with integral time.

### 3.7 Selectivity of the optical dual sensor

**Fig. 10:**
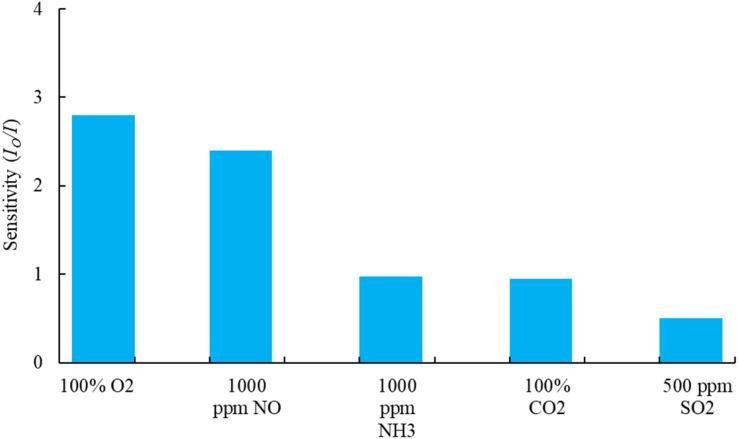
Selectivity effect of the dual sensor under different concentrations of gases.

Selectivity plays a critical role in maintaining the sensing performance of the optical dual sensor. To test the feasibility of the proposed sensor in practical applications, we carried out a selectivity test on different gas molecules of interest. In this work, the testing for each gas is carried out separately and alternately. Each gas test is performed for 20 minutes to monitor the gas response (change in fluorescence intensities) to the optical device sensor. Fig. 10, the sensor exhibited the highest response (2.8) to O_2_ gas molecules at the concentration of 100%, as well as the highest responses of NO (2.4) at 1000 ppm, NH_3_ (0.98) at 1000 ppm, CO_2_ (0.95) at 100%, and SO_2_ (0.5) at 500 ppm concentrations. All the purchased gases (mixed with N2 gas) were exposed to the sensor sample. Therefore, it is evident that CsPbBr_3_ QDs and FAPbI_3_ QDs molecules are highly selective to nitric oxide and oxygen molecules and could be used as an effective optical dual gas sensor in healthcare, biological, and biomedical applications.

## 4 Conclusions

This research is a new approach to the gas sensing technology of ratiometric optical dual gas sensors. The sensor fabrication and performance results of nitric oxide and oxygen sensitivity embedded in an ethyl cellulose matrix and silicone matrices using sensitivity dyes CsPbBr_3_ perovskite QDs and FAPbI_3_ perovskite QDs on filter paper membranes have been demonstrated successfully. The experimental result shows that the sensitivities of the ratiometric optical dual gas sensors for nitric oxide and oxygen are 2.4 and 2.8, respectively. The Stern–Volmer plots show linear relationships with nitric oxide and oxygen concentrations in the tested range of 0 to 1000 ppm and 0 to 100%, respectively. In addition, nitric oxide response and recovery times were 67 s and 69 s, respectively. The oxygen sensors were 66 s and 68 s, respectively. Thus, the sensitivity and selectivity of the proposed optical dual sensor suggest the feasibility of the simultaneous sensing of nitric oxide and oxygen. In conclusion, this research advances optical gas sensing technology, offering a versatile platform for sensing technology in healthcare, biological, and biomedical applications.

## Ethical statement

This study did not involve human participants or animals, and no ethical approval was required. All research procedures adhered to relevant ethical guidelines and best practices for non-human and non-animal research.

## Data availability

The necessary data used in the manuscript are already present in the manuscript.

## Author contributions

Rispandi: data curation, formal analysis, funding acquisition, investigation, software, visualization, writing – original draft. Cheng-Shane Chu: conceptualization, funding acquisition, methodology, project administration, resources, supervision, validation, writing – review & editing. Sri Nugroho: conceptualization, funding acquisition, methodology, project administration, resources, supervision, validation, writing – review & editing. Muhammad Imam Ammarullah: conceptualization, funding acquisition, methodology, project administration, resources, supervision, validation, writing – review & editing.

## Conflicts of interest

The authors declare that they have no known competing financial interests or personal relationships that could have appeared to influence the work reported in this paper.
